# A checklist recipe: making species data open and FAIR

**DOI:** 10.1093/database/baaa084

**Published:** 2020-11-11

**Authors:** Lien Reyserhove, Peter Desmet, Damiano Oldoni, Tim Adriaens, Diederik Strubbe, Amy J S Davis, Sonia Vanderhoeven, Filip Verloove, Quentin Groom

**Affiliations:** Research Institute for Nature and Forest (INBO), Havenlaan, B-1000 Brussels, Belgium; Research Institute for Nature and Forest (INBO), Havenlaan, B-1000 Brussels, Belgium; Research Institute for Nature and Forest (INBO), Havenlaan, B-1000 Brussels, Belgium; Research Institute for Nature and Forest (INBO), Havenlaan, B-1000 Brussels, Belgium; Terrestrial Ecology Unit, Ghent University, Karel Lodewijk Ledeganckstraat, 35, B-9000 Ghent, Belgium; Terrestrial Ecology Unit, Ghent University, Karel Lodewijk Ledeganckstraat, 35, B-9000 Ghent, Belgium; Belgian Biodiversity Platform, WTC III, Boulevard Simon Bolivar 30, Brussels, Belgium; Meise Botanic Garden, Nieuwelaan 38, B-1860 Meise, Belgium; Meise Botanic Garden, Nieuwelaan 38, B-1860 Meise, Belgium

## Abstract

Species checklists are a crucial source of information for research and policy. Unfortunately, many traditional species checklists vary wildly in their content, format, availability and maintenance. The fact that these are not open, findable, accessible, interoperable and reusable (FAIR) severely hampers fast and efficient information flow to policy and decision-making that are required to tackle the current biodiversity crisis. Here, we propose a reproducible, semi-automated workflow to transform traditional checklist data into a FAIR and open species registry. We showcase our workflow by applying it to the publication of the Manual of Alien Plants, a species checklist specifically developed for the Tracking Invasive Alien Species (TrIAS) project. Our approach combines source data management, reproducible data transformation to Darwin Core using R, version control, data documentation and publication to the Global Biodiversity Information Facility (GBIF). This checklist publication workflow is openly available for data holders and applicable to species registries varying in thematic, taxonomic or geographical scope and could serve as an important tool to open up research and strengthen environmental decision-making.

## Introduction

Despite the numerous organizations investing in biodiversity data gathering, it is recognized that valuable data can often not be fully utilized or reused (1, 2). Data may be accessible on the internet, but not necessarily machine readable, accessible in their entirety, licensed for reuse, easy to find, combine or repurposed. This greatly hampers the fast and efficient information flows to policy and decision-making that are required to tackle the current biodiversity crisis (3). This has led to the development of the findable, accessible, interoperable and reusable (FAIR) Principles for data (4). The founding principles of FAIR data are findability (F), accessibility (A), interoperability (I) and reusability (R) by both humans and computers. In short, the FAIR principles include a set of guidelines for the documentation and publication of data and metadata, the use of persistent identifiers, international standards and vocabularies, licensing and attributing provenance. The FAIR principles are inspired by Open Science but are not necessarily the same. While Open Science encompasses the free use of (meta)data and software (5), the FAIR principles do not describe the moral or ethical issues related to the openness of the data (6). Although FAIR data are by definition accessible, this can be under well-defined conditions to safeguard personal privacy or competitiveness. The FAIR principles not only apply to data and metadata in the conventional sense, but also to the tools and workflows that lead to the generation of the data (4). Source code required for data transformation, intermediate results and project planning are all elements to be shared and essential components of reproducible science (7).

Species checklists are lists of taxa known to occur in a given geographical area and period. They have a long tradition in biology as a means to summarize and communicate biogeographic and other information. Such annotated species lists are considered to be carefully reviewed, authoritative tools that provide a benchmark for decision-making in conservation of biodiversity. For example, through the use of checklists, it is possible to monitor and/or quantify the decline in pollinators (8), the threats to rare species (9) and emergence and trends of invasive alien species (10) over time. By giving an overview of biodiversity in an area, species checklists can help ensure the efficient allocation of conservation resources. Applying the FAIR principles to species inventories is a big departure from the traditional approach. Though they roughly follow a similar concept, species checklists vary widely in their content and format, ranging from paper-only versions published in books to structured digital files. Despite the fact that some checklists are the antithesis of FAIR, there are aspects of the traditional publication workflow that are worthy of preservation. Their medium of publication makes them accessible to local naturalists; they can be a motivating tool for biodiversity observers and the data are best maintained close to their source. Therefore, we seek methods to preserve the original checklist format while making them more widely accessible via publication on freely available internet repositories accompanied by clear documentation about how the checklists were originally prepared and then digitally transformed. For this reason, it is also important to incorporate a collaborative approach between the author and the party responsible for the online publication (i.e. the data publisher), so that the unique local and taxonomic context of the checklist can be addressed during the publication process.

A modern species inventory should be made available in an open data repository with a permissive license, use an internationally recognized data standard and be described with rich metadata. The Global Biodiversity Information Facility (GBIF, https://www.gbif.org/) is a prominent example of an international network and research infrastructure aimed at publishing open access, standardized biodiversity data. It has global coverage, which is important because data on biodiversity are frequently not housed in the country of origin of the species. GBIF makes use of community-developed (meta)data standards to ensure that data and metadata are machine readable (11). This provides standardization in the form of controlled vocabularies for some descriptive fields (http://rs.gbif.org/vocabulary/gbif) and enforces the use of standard licenses to make data reusable (https://www.gbif.org/terms). GBIF also provides harvesting and publishing tools (12). Open publication of checklist data on GBIF allows the integration of checklists from all over the world, which is the basis for accurate and up-to-date data on species distributions, while maintaining provenance and ensuring visibility of the original work (13, 14).

Since an author writing a checklist might use local field names and denominators, standardization is needed to make the data interoperable worldwide. One important standard for sharing biodiversity data is the Darwin Core (DwC) standard (11), a global standard developed by Biodiversity Information Standards (TDWG) and adopted by GBIF. DwC provides a glossary of terms developed to share and integrate checklist data by providing identifiers, vocabularies and definitions (15). A Darwin Core Archive (DwC-A) is a set of one or more structured and standardized data files generated from the source data, supplemented with an Extensible Markup Language (XML) metadata file that describes its content (https://dwc.tdwg.org/text/). The process of translating the original fields in the source files to the appropriate DwC terms is called ‘mapping’. The workflow behind the mapping process is often labor-intensive and unreproducible due to poor documentation of the transformation steps. However, this can be overcome by the use of automated mapping scripts using open software tools (such as R or Python) to establish reproducible and efficient workflows. Computational reproducibility is the ability to exactly reproduce results given the same data (16). It can greatly increase productivity as less time is wasted to confirm results, to test software updates, or to recover lost outputs. Reproducibility can only be successful when the mapping code is accompanied by sufficient documentation to understand it. In ‘literate programing’ (17), the computer-readable source code is accompanied by a narrative explaining its logic in a natural language, such as English. By developing a human and machine-readable script to transform source data to a DwC-A format, the process of checklist publication can be greatly improved: once a data publisher has created the data mapping, they do not have to start from scratch if the source data has been updated. The mapping script can be run again, with minor modifications when necessary. By changing the source data (in case of updates) and/or the mapping scripts, the generated DwC files will automatically be altered.

**Figure 1.**
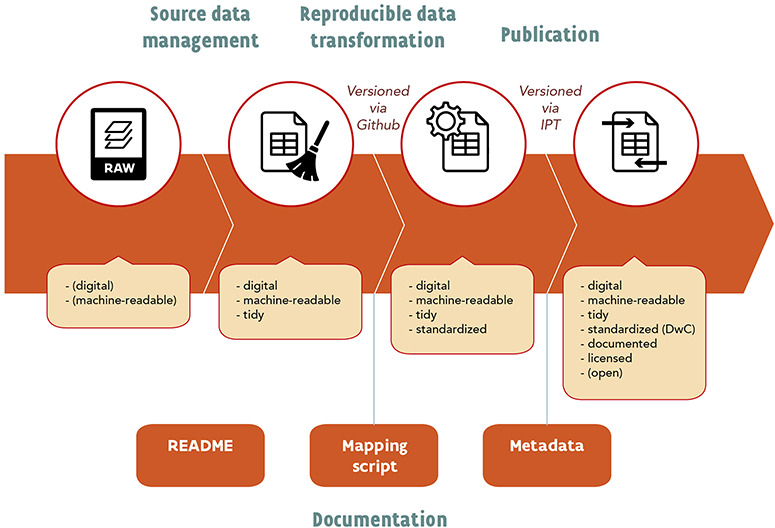
Schematic overview of the suggested workflow.

Here we propose a holistic workflow for checklist publication: one that is open, reproducible and versioned and combines data standardization to DwC with data publication on GBIF (Figure 1). This workflow is a stepwise process and includes (i) source data management to produce ‘tidy data’, (ii) automated and reproducible data transformation to produce interoperable data, (iii) data documentation and (iv) data publication to produce FAIR and open data. Each of these components is under version control. Below, each step in the workflow is discussed separately, using the publication of the Manual of the Alien Plants of Belgium (18)—a checklist of non-native species—as an example.

## Checklist publication workflow

### Step 1: Source data management

Data management, the practice of collecting, processing, analyzing, storing and sharing, is fundamental to the success of any project. Done efficiently, any researcher should be able to contribute or repeat the project and to interpret the data without assistance of the original project partners. It all starts with the raw source data. The raw data may be collected or exist as hand-written notes, printed text, images, digital non-tabular text or computer-readable spreadsheets. The reproducible data transformation step in our workflow requires the use of a digital and machine-readable format as input. Raw data existing in analog or non-text form should thus be digitized and made available as structured data first. To better structure source data from the start, GBIF provides templates to facilitate data entry by data holders (https://www.gbif.org/dataset-classes). The fewer agents involved in the transformation of the raw dataset to the digital, machine readable format, the better. A data publisher receiving or getting access to source data should treat it as read-only, i.e. raw data should stay raw. All required data handling should be realized in the reproducible data transformation step, explained below. An exception can be made for ‘structural’ organizations of the source data that would vastly improve its management and processing. The general rule is: the more straightforward, uncomplicated and automated the workflow, the easier, faster and more robust the process of repeating it will be. Ideally, the dataset should conform to the Tidy Data Principles (19). These are a set of recommendations to organize your data within a dataset. They have been developed to facilitate data exploration, processing and analysis. The three characteristics that define Tidy Data are: (i) each variable forms a column, (ii) each observation forms a row and (iii) each type of observational unit forms a table. Messy or untidy data are any other arrangement of the data. By making datasets Tidy, it reduces ambiguity for both humans and computers and this clarity reduces the potential for errors in understanding and processing. In case of messy source datasets, the best option is to consult with the dataset author to improve the quality of the source data. When this is not possible, one should create an intermediate, Tidy Data product and document all structural changes that have led to its generation.

### Step 2: Reproducible data transformation

The core part of the reproducible workflow is the mapping script: a documented script to transform the (preferably) Tidy Dataset to one or more standardized DwC files. A DwC checklist constitutes a delimited text file containing the taxonomic list: the taxon core (http://rs.gbif.org/core/dwc_taxon_2015-04-24.xml). Each line in this core file refers to a single taxon, with information related to higher classification, synonymy and rank. The first column in this taxon core contains a unique key linked to that specific taxon: the taxonID. This is a required DwC term and serves as a unique identifier for a taxon in that specific checklist. Ideally, this identifier should be persistent and globally unique, but should at least be unique within the published dataset. The taxonID makes it possible to relate information to that specific taxon, such as distribution information, taxon descriptions or vernacular names. This type of information can be shared in dedicated extension files (http://rs.gbif.org/extension/dwc/), where each row (one or multiple) relates to a taxonID. The entire setup allows to relate information about a taxon in one-to-many relationships or star schema (11).

For our data transformation workflow, we use R as a programing language and R Studio (http://www.rstudio.com) as a development environment, although other (ideally open-source) programing languages and environments are equally fit. The mapping script is usually divided into four different sections to structure the mapping process: read source data, preprocessing, DwC mapping and post-processing. Before reading the source data, we load the ‘packages’ required for the data transformation. R has an impressive number of ‘packages’ that have been built by the community and can be installed easily. In this respect, the Tidyverse packages (20) are a collection of packages designed for everyday data transformation and are thus highly suitable for the transformation of source data to DwC. The core packages work well together and share the same philosophy, grammar and data structure. The most important Tidyverse functions used in the DwC mapping process are mutate() to update or add a column, recode() to change values in a column and case_when() to change values in a column based on conditional statements. The goal of the preprocessing step is to clean and prepare the dataset for the subsequent mapping. This includes small structural changes such as removing empty rows or columns, or adding extra columns as an intermediate product to restructure the original content. This might be necessary when information captured in multiple columns of the input dataset must be combined into a single DwC term. In the next section, the DwC mapping section, the DwC Taxon files are generated. This process is sequential by nature: we first generate the taxon core file, followed by the extension files. For each file (core or extension), we use a series of iterative mapping steps to transform the dataset into a standardized DwC file. In each mapping iteration, we evaluate the DwC standard’s terms (see https://dwc.tdwg.org/terms/for a complete overview) for an appropriate fit with one or more fields in the input dataset. Whenever there is a match, we add the DwC term to the dataset using the Tidyverse functions. The file is thus generated by adding the DwC terms one by one. In the mapping process, we can distinguish between three different types of 6generated DwC terms, based on its relation to source data:

**Static DwC terms:** terms with a fixed value for every record in the dataset, i.e. their content is the same for the whole dataset. This is the case for most metadata terms (also known as record-level terms) in the taxon core, such as datasetName or license. These terms are generated using the mutate() function.
**Unaltered DwC terms:** terms for which the content of the field is an exact, unaltered copy of the corresponding field in the input data. These terms are generated using the mutate() function.
**Altered DwC terms:** terms for which the content of the DwC term is a transformation of one or more specific fields in the source data. This is the case when the original data needs to be mapped to a vocabulary or other standard. These terms are generated using the mutate() function combined with recode() or case_when(). Several DwC terms require the use of a specific formats or controlled vocabulary values: ISO 3166 for names of countries and their subdivisions (DwC terms locality and countryCode), ISO 8601 for date and time information (DwC term eventDate), vocabularies defined by GBIF (http://rs.gbif.org/vocabulary/gbif/) (DwC terms such as occurrenceStatus, establishmentMeans or taxonRank) and standards defined by Biodiversity Information Standards (TDWG), such as the World Geographical Scheme for Recording Plant Distributions (WGSRPD) (21) for native range information.

By adding the DwC terms to the input dataset instead of creating a new, separate dataset, we keep the link between the original columns and the mapped DwC terms. Each mapping iteration adds a new column to the dataset until all relevant DwC terms have been mapped. The list of DwC terms is long and choosing the correct term can be challenging. Resources such as the Global Names Architecture Profile (15), DwC Hour (https://www.id-igbio.org/content/darwin-core-hour-webinar-series), DwC QA (https://github.com/tdwg/dwc-qa) or the data publication guidelines of the Research Institute for Nature and Forest (INBO) (https://github.com/inbo/data-publication) can provide guidance. In the post-processing section, all original columns of the input dataset are removed and only the mapped DwC terms remain. These are then exported as a structured csv file. To summarize, the mapping script transforms the source data term-by-term into a DwC taxon core and extension files. The script can be re-run, reviewed and improved as needed.

### Step 3: Documentation

Clear documentation is an essential aspect of the work-flow. We use R Markdown (https://github.com/o combine narrative text and emrstudio/rmarkdown), a file format used to combine narrative text and embedded R code (22), to describe and execute the procedural standardization steps to DwC. This form of literate programing allows the clarification of decisions, an increase in transparency and collaboration, as well as easier tracing of mistakes or bugs in the code. In addition to workflow documentation, detailed dataset documentation is needed to provide contextual information about the checklist. To publish the checklist on GBIF, metadata needs to conform to the GBIF Metadata Profile (GMP), an extension of Ecological Metadata Language (EML) (23): a standard to record information about ecological datasets in XML. This profile includes information related to the publisher, authors, keywords and geographic, taxonomic and temporal scope of the dataset, as well as project and sampling information, the latter of which can be used to document source data provenance and data transformation workflow. Finally, it specifies the license of the dataset, which can be one of three options supported by GBIF: the Creative Commons Attribution license (https://creativecommons.org/licenses/by/4.0/), the Creative Commons Attribution non-commercial license (https://creativecommons.org/licenses/by-nc/4.0/) and the Creative Commons Zero waiver (https://creativecommons.org/publicdomain/zero/1.0/). The resource metadata can be filled in the built-in metadata editor of the GBIF Integrated Publishing Toolkit (IPT) (12). This metadata editor then automatically transforms the metadata to an EML file. However, to facilitate collaborative working, we recommend a shared Google Docs or similar to draft and review the metadata, and then copying this information to the IPT.

To ensure that the checklist can be understood, regenerated and re-used in the long term, good organization of the data files is essential. Organization requires explicit and consistent naming of variables, files and repositories and the use of a clear folder hierarchy. For example, for each checklist, a dataset shortname should be defined at the start of the project as it is used as the name of the GitHub repository, name of the resource in the IPT, unique identifiers such as the taxonID, and some file names. The structure and relationships between all files should be described in a descriptive README, which provides orientation for the project (Figure 2).

**Figure:**
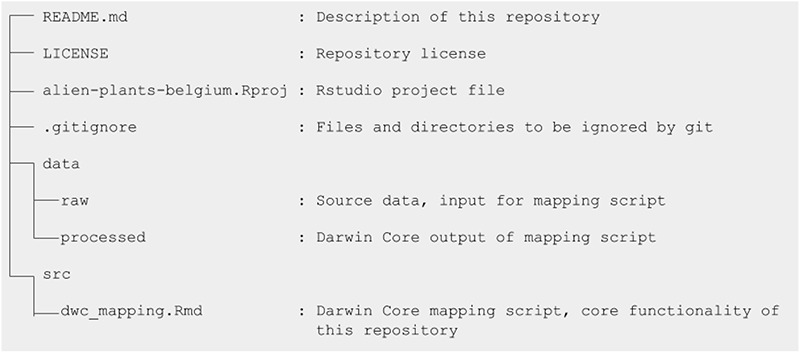
Structure of the GitHub repository of the Manual of Alien Plants Belgium.

### Step 4: Publication

The checklist is ready for publication once the source data have been standardized to DwC, the dataset documented with metadata, and both sufficiently reviewed by the authors. This can be done by creating a checklist resource on an IPT, ideally one hosted by a trusted data hosting center (https://www.gbif.org/data-hosting). The generated DwC files should be uploaded and mapped to their appropriate core or extension type, which can be installed by the IPT administrator if not available. Since the files contain DwC terms as column headers, they will be recognized by the IPT and auto-mapped. If written elsewhere, metadata should be copied to the appropriate sections of the IPT metadata editor. Once ready, the resource can be set to public and published. The IPT will then create version 1.0 of the dataset, and bundle data and metadata into a publicly available DwC-A. At this point, the dataset is open and according to FAIR principles, except for findability.

To increase findability, the dataset should be registered with GBIF. This can be done from the IPT, once the organization affiliated with the first author or an organization acting as custodian is an endorsed data publisher (https://www.gbif.org/become-a-publisher). Registering with GBIF will trigger a number of things: the dataset will be added to the GBIF registry, its metadata will be made fully searchable and (if one is not associated already) a Digital Object Identifier (DOI) will be created at DataCite as a unique identifier for the dataset, resolving to a GBIF.org dataset page. This makes the metadata findable and fully adhering FAIR principles. But this will be done for the data as well. Since these are standardized to DwC, GBIF can and will harvest these, match taxa to the GBIF Backbone Taxonomy (24) and integrate distribution, vernacular name and description information into species pages at GBIF.org (see, e.g. https://www.gbif.org/species/5415453). The published checklist is not only open and FAIR, but these properties are immediately put to use to have it contribute to the public knowledge of species on earth.

### Version control

To keep track of changes to the workflow, trace back issues and allow review of changes, all relevant documents can be put under version control: a structured and transparent means of tracking changes. The GBIF IPT allows for version control of the published data (and its metadata) and Google Docs allows for version control (and collaboration) of metadata documents. To version and document code (the mapping script), we suggest using GitHub (http://www.github.com). GitHub is a popular, web-based, software development platform, allowing users to remotely collaborate and publish software code and documentation. The version-controlled files are all stored remotely in a public ‘repository’, which has a README file to orient potential users or collaborators regarding the purpose of the project and structure of the repository. This repository can be downloaded to set up a local version (local repository) on your computer, which allows you to write code and upload files to the workflow. Local changes can be regularly ‘committed’ and ‘pushed’ to the remote repository. To facilitate collaborative working Git allows you to set up ‘branches’ when developing and testing some features (e.g. changes in metadata or mapping). When done, a pull request is created to propose the changes. Once they are positively evaluated by one or more collaborators they can be merged and be part of the ‘master’ or ‘default branch’. Each commit, branch and pull request is integrated in the version history of the project and can be consulted any time in the workflow. Additionally, the GitHub repository hosts an issues page for reporting software bugs, asking general questions or proposing enhancements. For scientists unfamiliar to version control with Git and GitHub, see Blischak *et al.* (25) for an introduction.

### Conclusion

**Figure 3.**
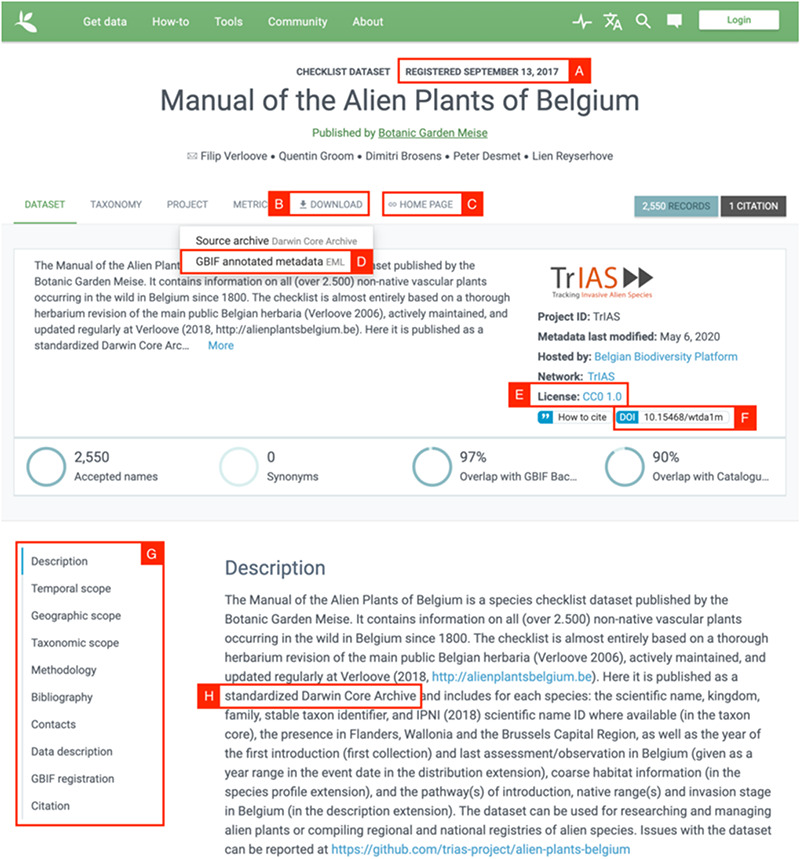
Screenshot of the GBIF dataset page for the Manual of Alien Plants Belgium. Letters A-H refer to the different aspects of FAIR data, see text for further details.

The end product of the checklist publication workflow is a dataset that is openly available and complies with the FAIR principles. It is ‘Findable’ by its globally unique and persistent identifier (DOI, Figure 3F), described with rich metadata (Figure 3G) and registered in GBIF (Figure 3A), ‘Accessible’ by simply clicking on the download link provided in GBIF (Figure 3B), ‘Interoperable’ as it uses a broadly applicable biodiversity standard and vocabularies provided by TDWG and GBIF (Figure 3D, H), ‘Reusable’ as it is associated with detailed provenance (Figure 3C) and released with a clear data usage license: the open Creative Commons license (Figure 3E). In addition, the whole ‘workflow’ for creating this dataset is FAIR and open as well, and placed under version control, to increase transparency and collaboration.

## Case study: Manual of the Alien Plants of Belgium

We provide proof of concept of our stepwise data publication workflow using the publication of the Manual of the Alien plants of Belgium (18). This species checklist, published by the Meise Botanic Garden, is an authoritative checklist integrating all alien plants recorded in Belgium since 1800. The Manual of Alien Plants of Belgium is not merely a list of scientific names; for each species, it includes information regarding taxon rank, higher classification, mode of introduction, date of first and last observation, origin, occurrence status in the three different regions of Belgium (Flanders, Wallonia and the Brussels-Capital Region), degree of naturalization, vector of introduction and habitat. The dataset is publicly available (downloadable from the website http://alienplantsbelgium.be/), in a proprietary and Tidy format (Microsoft Excel). The website includes all consulted sources to assure full provenance. In this example, we start the mapping process using the penultimate version of the checklist, which enables us to demonstrate how it can be updated for the latest version.

As the first step in the publication workflow of this checklist, we created a public GitHub repository to manage, share and organize our work (https://github.com/trias-project/alien-plants-belgium). We gave it the dataset short-name alien-plants-belgium and host it under the TrIAS project organization page on GitHub (https://github.com/trias-project), which hosts, among others, all repositories related to checklist publication of alien species within the TrIAS project (26) (http://trias-project.be). The repository has a clearly defined file structure, which is based on the Cookiecutter Data Science template (http://drivendata.github.io/cookiecutter-data-science), and basically includes a concise README, an open-source software license, a data directory to upload the input and processed datasets and a src folder to contain the DwC mapping script (Figure 2): a R Markdown file called dwc_mapping.Rmd. Before mapping, we also set up the repository as an R Studio project, creating an alien-plants-belgium.Rproj file in the root directory. Opening this file would open a new R Studio session with the root directory as a working directory, allowing all contributors to use the same relative paths. To work on the repository on a local machine, a project collaborator would clone it and create a branch for their changes. These changes were generally committed in logical chunks and pushed to GitHub at the end of the day, safeguarding them from loss. Once the changes were ready for review, a pull request was created and reviewed by another project collaborator. Requested changes were incorporated and once approved, incorporated by merging these into the master branch. Anyone could then pull those to their machine to get the latest version.

**Table 1.** Overview of the relation between the source field and the matching Darwin Core term, with examples from the Manual of Alien Plants Belgium. For each of the Darwin Core terms, the related Darwin Core file is given, together with an example of the mapping and the matching vocabulary (when applicable)

Darwin Core file	Darwin Core term	Based on source field	Example	Vocabulary
taxon	language		en	ISO 639–1
taxon	license		http://creativecommons.org/publicdomain/zero/1.0/	
taxon	rightsholder		botanic garden meise	
taxon	datasetID		https://doi.org/10.15468/wtda1m	
taxon	datasetname		manual of the alien plants of belgium	
taxon	taxonID	taxon	alien-plants-belgium:taxon:a65145fd1f24f081a1931f9874af48d9	
taxon	scientificnameID	scientificnameID	http://ipni.org/urn:lsid:ipni.org:names:44920-1	
taxon	scientificName	taxon	acanthus spinosus L.	
taxon	kingdom		plantae	
taxon	family	family	acanthaceae	
taxon	taxonrank	taxonrank	species	
taxon	nomenclaturalCode		ICN	
distribution	taxonID	taxon	alien-plants-belgium:taxon:a65145fd1f24f081a1931f9874af48d9	
distribution	locationID	presence_Fl, presence_Br, presence_Wa	ISO_3166-2:BE-VLG	ISO 3166
distribution	locality	presence_Fl, presence_Br, presence_Wa	flemish region	
distribution	countryCode		BE	ISO 3166
distribution	occurrenceStatus	presence_Fl, presence_Br, presence_Wa	present	http://rs.gbif.org/vocabulary/gbif/occurrence_status.xml
distribution	establishmentMeans		introduced	http://rs.gbif.org/vocabulary/gbif/establishment_means.xml
distribution	eventDate	FR, MRR	2016/2018	ISO 8601
speciesprofile	taxonID	taxon	alien-plants-belgium:taxon:a65145fd1f24f081a1931f9874af48d9	
speciesprofile	isMarine		FALSE	
speciesprofile	isFreshwater	habitat	FALSE	
speciesprofile	isTerrestrial	habitat	TRUE	
description: native range	taxonID	taxon	alien-plants-belgium:taxon:a65145fd1f24f081a1931f9874af48d9	
description: native range	description	origin	Europe (WGSRPD:1)	(21)
description: native range	type		native range	
description: native range	language		en	
description: pathway	taxonID	taxon	alien-plants-belgium:taxon:a65145fd1f24f081a1931f9874af48d9	
description: pathway	description	V/I	cbd_2014_pathway:escape_horticulture	(26)
description: pathway	type		pathway	
description: pathway	language		en	
description: invasion stage	taxonID	taxon	alien-plants-belgium:taxon:a65145fd1f24f081a1931f9874af48d9	
description: invasion stage	description	D/N	casual	
description: invasion stage	type		invasion stage	(27)
description: invasion stage	language		en	

The source dataset (Checklist5.xlsx) was downloaded from the website and uploaded to the data/raw folder in the GitHub repository. This checklist served as the input data file for the data transformation to DwC in R Studio. The dwc_mapping.Rmd script was subdivided into four different sections to structure the mapping process. The pre-processing step was used to generate a globally unique taxonID as the main taxon identifier, since the numerical identifier used in the source checklist was not globally unique and prone to shift when inserting taxa. This new taxonID was created using a combination of the dataset shortname (alien-plants-belgium), a reference to the taxon core (taxon) and an alphanumeric code based on the combination of the taxon’s scientific name and kingdom. For example, the taxonID of *Acanthus mollis L.* is alien-plants-belgium:taxon:509ddbbaa5ecbb8d91899905cfc9491c. In the next section, data are mapped to DwC as four files: one taxon core and a distribution, species profile and description extension (Table 1). The original content of the input dataset was translated to a DwC term whenever we found a match, either as a static, an unaltered or altered term (Table 1). An extra pre-processing step was needed in the subsection dedicated to the mapping of the distribution extension, to facilitate interpretation and mapping of the location and date information. Three fields in the source data had no matching DwC term and were integrated in the description extension: origin, degree of naturalization (D/N in the raw dataset) and vector of introduction (V/I in the raw dataset). For each of these variables, we specified the type of description in the DwC field type (respectively: native range, degree of establishment and pathway of introduction), and its associated value in the corresponding DwC field value. Several DwC terms were mapped to a standard: ISO 639–1 for language, ISO 3166 for countryCode and locationID, ISO 8601 for eventDate, GBIF vocabularies for taxonRank, nomenclaturalCode, occurrenceStatus and establishmentMeans, TDWG vocabulary for native range (21) when applicable. To express the pathway of introduction and degree of establishment in a standardized way, we used controlled vocabularies that are globally adopted by the invasion biology community: CBD (27) for pathways and Blackburn *et al.* (28) for degree of establishment (29). DwC files were generated as csv files to the data/processed folder. The processed DwC files were then uploaded to the IPT instance of the Belgian Biodiversity Platform (https://ipt.biodiversity.be/resource?r=alien-plan-ts-belgium). The data were supplemented with metadata using the IPT metadata editor, which automatically generated an EML file. Once reviewed and ready, a first version of the dataset was then published on the IPT (making it publicly available there) and registered with GBIF. GBIF then harvested the checklist and created a DOI (https://doi.org/10.15468/wtda1m). This DOI was used as a stable identifier for the checklist and updated in the field datasetID of the taxon core. For this update to become visible on GBIF, the dataset was re-published.

Each time a new release of the checklist is available, a project collaborator can create a new branch (e.g. https://github.com/trias-project/alien-plants-belgium/tree/update-2020-05-06 for the most recent update), upload the latest version of the checklist to the data/raw folder and re-run the mapping script to generate the updated DwC files, which are then automatically uploaded to the data/processed folder. These changes are then submitted as a pull request (https://github.com/trias-project/alien-plants-belgium/pull/80) and reviewed before incorporating. A pull request visualizes which and what sections of files have changed, hiding sections that have not changed. For instance, an update in the date of first record (FR) of an existing taxon in the raw dataset will result in the deletion of the line in the distribution extension with that specific eventDate information (indicated in red) and the generation of a new line with the updated eventDate information (indicated in green). By reviewing the changes caused by the update, we can then decide whether or not small changes in the mapping script are required. For instance, a new value in the source data should be mapped to a controlled vocabulary value. The updated DwC files can then be uploaded to the IPT where we can also update the metadata section if required. Each new version of the dataset on the IPT can be consulted (https://ipt.biodiversity.be/resource?r=alien-plants-belgium&v=1.9). The last step is then to republish the dataset on GBIF.

## Discussion

The workflow described above transforms a raw, unstandardized dataset to a FAIR and open dataset published on GBIF (Figure 3). The mapping process is entirely repeatable, but the full publication workflow does require some manual steps. The generated DwC files have to be uploaded to the IPT, which could be improved by allowing the IPT to fetch data from a URL. Metadata too are copied to the IPT from an environment that allows easier collaboration (Google Docs). Research repositories in general seem to struggle with offering good collaboration tools for metadata, while ensuring these are standardized. The checklist data itself can generally be well standardized to DwC, but some specific information could have been mapped better if a suitable DwC term or controlled vocabulary was available. The DwC standard is under active maintenance and evolves to meet the changing needs of biodiversity informatics (30). For example, suggestions were made to improve the standard for reporting on the pathway of introduction, degree of establishment and status of alien species (29).

To lower the barrier for data owners to publish their data using the method described in this paper, we developed a ‘checklist recipe’ (31), which won the 2018 Ebbe Nielsen Challenge. The recipe is a template GitHub repository, specifically developed to assist data holders in standardizing species checklists to DwC using R. It is based on the experience we gained by publishing checklist data for the TrIAS project, including the Manual of the Alien Plants of Belgium presented in the case study. The basic ingredients for this recipe are (i) a template spreadsheet with a list of predefined fields covering both taxonomic and distribution information, (ii) a template mapping script to transform the data to DwC and (iii) a wiki describing how to use these template documents. One can also upload their own source data file and/or adapt the mapping script to publish GBIF occurrence or sampling-event datasets (http://www.gbif.org/dataset-classes). By providing the data providers with the necessary tools, tips and methods on how to maintain and publish their dataset, we empower them to publish their own dataset according to best practices.

The approach we bring forward here differs from traditional species registry initiatives, where experts are asked to contribute information to a centralized database, which is set up for a specific taxonomic, geographic or thematic scope. For alien species for example, such initiatives include DAISIE (32) and EASIN (33, 34) (https://easin.jrc.ec.europa.eu/easin) at the European level and NOBANIS (35) (https://www.nobanis.org/) and ESENIAS (36, 37) (http://www.esenias.org/) at the regional level. These initiatives often depend on temporary project funding and can face sustainability problems (e.g. DAISIE and NOBANIS). With the exception of DAISIE which was recently published as an open and FAIR dataset (38), the data these initiatives collect are lost when their infrastructure is no longer maintained. By allowing experts to publish their checklist using widely adopted standards and infrastructure, they have more control over the tools they use and when to publish, they get more credit, and their work is less likely to be lost. By making the standardization process repeatable and publicly available, it is also easier for others to contribute to or reuse the work, or to transfer maintenance when necessary. This makes publishing checklist data more cost-efficient and sustainable.

Since these checklists often have a limited taxonomic, geographic or thematic scope, they should be consolidated to effectively support research and policy. This process is greatly facilitated by making checklists open and FAIR: scientific names from checklists published to GBIF are automatically matched to the GBIF Backbone Taxonomy (24). By harvesting these interoperable checklist data using the GBIF Species Application Programming Interface (API) (https://www.gbif.org/developer/species), it is possible to create a unified (e.g. national) checklist in an automated, transparent and repeatable way. Such an approach has been adopted to create a unified checklist of alien species in Belgium (39). This unified checklist, which is based on 9 authoritative checklists published through the repeatable process we described, was accepted as the Belgian contribution to the Global Register of Introduced and Invasive Species (14). We hope it inspires others to do the same.
